# RETRACTION: “Sublethal Heavy Metal Stress Stimulates Innate Immunity in Tomato”

**DOI:** 10.1155/tswj/9874191

**Published:** 2026-08-18

**Authors:** The Scientific World Journal

RETRACTION: N. Chakraborty, S. Chandra, and K. Acharya, “Sublethal Heavy Metal Stress Stimulates Innate Immunity in Tomato,” *The Scientific World Journal,* no. 2015 (2015). https://doi.org/10.1155/2015/208649.

The above article, published online on 2 February 2015 in Wiley Online Library (wileyonlinelibrary.com), has been retracted by agreement between the journal’s Academic Editor , Ricardo Aroca; and John Wiley & Sons Ltd.

The retraction has been agreed following an investigation of the concerns raised by *Salix pulchra* on PubPeer [1], which identified an instance of figure duplication.

More specifically, the images of DAF‐2DA staining of control and 1mM CuCl_2_ ‐ treated leaf petiole sections in Figure 2 share overlapping features.

As a result of the investigation, the data and conclusions of this article are considered unreliable.

The authors disagree with this retraction.
